# Short-Term Effects of Complex Training on Agility with the Ball, Speed, Efficiency of Crossing and Shooting in Youth Soccer Players

**DOI:** 10.2478/hukin-2014-0095

**Published:** 2014-11-12

**Authors:** Braulio Cavaco, Nelson Sousa, Victor Machado dos Reis, Nuno Garrido, Francisco Saavedra, Romeu Mendes, José Vilaça-Alves

**Affiliations:** 1 Sport Sciences, Exercise and Health Departament, University of Trás-os-Montes e Alto Douro, Vila Real, Portugal.; 2 Research Center in Sports Sciences, Health Sciences and Human Development - CIDESD, Vila Real, Portugal.

**Keywords:** strength, post activation potentiation, soccer

## Abstract

Complex training (CXT) is the result of a combination of strength and plyometric exercises in the same session. This method has recently been used in the preparation of athletes of different sports. The aim of the present study was to observe the acute effects of a CXT program of 6 weeks: i) on agility with the ball, sprinting and the efficiency of crossing and shooting in youth soccer players; ii) and the influence of the number of CXT sessions per week (one vs. two). Sixteen youth male soccer players were randomly divided into three groups: a group that performed one weekly CXT session (GCT1, n = 5, age: 13.80 ± 0.45 years); or a group that performed two weekly CXT sessions (GCT2, n = 5, age: 14.20 ± 0.45 years); or a control group that did not perform the CTX (n = 6, age: 14.20 ± 0.84 years). All groups maintained their regular soccer training sessions. No significant interactions were found between GCT1 and GCT2 in all variables. Significant statistical differences were identified (F = 1139, p = 0.02, μ_p_^2^ = 0531) in the pre-test versus post-test, for both experimental groups, in shot effectiveness. In conclusion, the CXT program proved to be an effective method in boosting abilities and motor skills associated with soccer among young athletes, particularly in increasing shot effectiveness.

## Introduction

Complex training (CTX) alternates a high-load exercise with an explosive skill or plyometric exercise, set to set, in the same workout. This method is supported by the assumption of a post activation potentiation of the neuromuscular system. This phenomenon is defined by an increase of muscle contraction related to its previous contraction.

Recently, the CXT method has been successfully applied in various sports. In a study with rugby athletes, significant improvements in speed were identified after four training sessions (Comyns et al., 2010). Santos and Janeira (2008) found performance improvements, in young basketball players (14–15 year olds), at the level of thrust and strength of the upper limbs following a ten-week CXT program.

There has also been interest in introducing this method in soccer. Christou et al. (2006) suggested that strength training in addition to soccer practice may be beneficial to the overall development of youth athletes’ physical abilities. In this sense, Maio Alves et al. (2010), in a study of youth Portuguese soccer players, identified significant increases in speed and vertical jump performance, after six weeks of training independently of the number of training sessions per week (1 or 2). It is important to refer that these numbers of sessions are the ones used in Portugal in soccer training of athletes at this age group.

The CXT protocols usually used in sports training are based on the combination of Olympic powerlift movements with the technical specific skills of a particular sport discipline. Therefore, in the literature, other methods appear that usually differ in the type of exercise used (explosive, isometric or plyometric) and in the time between pairs of drills (without rest and with a rest interval between 3 and 18 minutes). Mostly, the rest periods between exercise pairs are important for the observation of post activation potential and these periods are separate and dependent on the level of strength and experience of the athlete (Young et al., 1988; Kotzamanidis et al., 2005; Tricoli et al., 2005; Kilduff et al., 2007; Santos et al., 2008). The need of prolonged rest periods between drill pairs affects negatively the dynamic of the training sessions. As some studies observed an increase in the performance by introducing a lower intensity plyometric exercise between pairs of drills (Maio Alves et al., 2010), it seems that we can perform the combination of the strength exercise, the sprint and a specific technical skill without rest and increase game performance.

Nevertheless, in the scientific literature, several chronic studies on the effects of CXT are presented, especially with different training protocols: i) without rest between the high-load exercise and the explosive skill; ii) with more focus laid on one of technical exercises between a pair of drills; iii) and with a different number of sessions per week. Therefore, this study aimed to test the effects of a six-week CXT program on agility, speed, and efficiency of crossing and shooting in youth soccer players; and to compare the effects of two weekly CXT sessions with one-weekly CXT session in addition to soccer training.

## Material and Methods

### Experimental Approach to the Problem

The short-term study with a repeated-measures design was used in order to determine the effects of CXT on agility, speed, and efficiency of crossing and shooting in youth soccer players. The subjects were randomized into 3 groups (2 experimental groups and 1 control group). A total of 8 weeks of the intervention were divided into an adaptation period of 2 weeks and a CXT program of 6 weeks applied to the experimental groups. Additionally, all groups performed their normal soccer training. The players were evaluated in a 15 m sprint, 15 m agility with the ball, crossing efficiency and shooting efficiency, at 2 moments (pre- vs. post-test). The first evaluation was carried before the CXT program and the second after 6 weeks.

### Subjects

Sixteen male adolescent soccer players were randomly divided into three groups: a group that performed one weekly CXT session (GCT1, n = 5, age: 13.80 ± 0.45 years); or a group that performed two weekly CXT sessions (GCT2, n = 5, age: 14.20 ± 0.45 years); or a control group that did not perform CTX (CONT, n = 6, age: 14.20 ± 0.84 years). Prior to the commencement of the research, the players, coaches and the guardians of the players included in the study were informed of the protocol and the hypothetical experimental risks. Afterwards, the guardians of the participants signed an informed consent form, elaborated according to the declaration of Helsinki. Table 1 presents the subjects characteristics at baseline.

### Procedures

All the youth soccer players performed a two week adaptation strength-training program with 3 sessions per week, in order to provide a base for the execution of the squat exercise and prevent possible injuries. In the beginning of the soccer training session, after a warm-up, 3 sets of 12 repetitions were carried out of the squat exercise.

After the adaptation period, the youth soccer players were submitted to the first evaluation in the following tests: 15 m sprint, 15 m agility with the ball, crossing efficiency and shooting efficiency. After this evaluation, the youth soccer players were randomized into 3 groups (2 experimental groups, GCT1 and GCT2 and 1 control group, CONT). The youth soccer players from the GCT1 and GCT2 were evaluated in one repetition maximum (1-RM) on the squat exercise in order to determine the CTX workload.

The subjects of the GCT1 and GCT2 performed their normal soccer training along with the CTX program (1 session per week for the GTC1 and 2 sessions per week for the GCT2). The CONT group performed only regular soccer training. All groups performed similar soccer training. The CTX was performed at the beginning of a soccer training session after a warm-up. Each training session was organized in 2 stations. The exercises in each station included: first station, 3 sets, with 6 squat repetitions at 85% of 1-RM, 15 m plus top speed and cross, separated by 3 min of rest; second station, 3 sets, 6 repetitions of the squat at 85% of 1-RM, plus agility with the ball and shot at the goal, separated by 3 min of rest. The load in the squat exercise was increased by 5% from initial 1RM each 2 weeks. After finishing the CTX program, all subjects were revaluated.

### Measurements

Maximum strength in the squat exercise was measured by the 1-RM test through the procedures suggested by Kraemer and Fry (1998). The evaluation was carried out after the two adaptation weeks. The soccer players of the GCT1 and GCT2 groups were always kept under surveillance of a professional with high experience in strength training.

Maximum speed was measured by the 15 m sprint test, using photocells (Speed Trap II -Browser Timing Systems).

**Figure 1:**
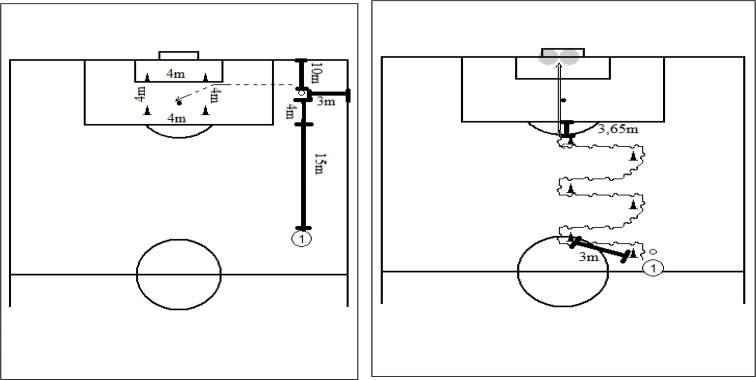
Straight line 15 m sprinting, crossing test, dribbling between the cones and shooting test. 1 – athlete

Crossing efficiency was measured through a cross to an enclosed 4 × 4 m area at the penalty mark. The ball had to cross the area delimited by traffic pylons, above the level of the crossbar, and the ball would move in the delimited area already on a downward path to be considered effective. The ball was situated 4 m from the last photocell, 3 m from the lateral line and 10 m from the finish line, and could only be played for cruising in the shortest possible time. This exercise was always performed in the right flank of the field game and the procedure was performed 3 times with a 3 min rest between sets. The best time and efficacy of the 3 crosses was registered for further analysis (Figure 1).

The agility test with the ball was used to verify the ability of the participants dribbling the cones while assessing time with a chronograph, as reported in other studies (McGregor et al., 1999). To evaluate agility with the ball plus shooting at the goal, 6 cones were diagonally placed with a distance between them of 3 m, centered on the goal. Participants had to dribble between the cones as fast as possible, without turning over the cones or losing control of the ball. The test was cancelled whenever the protocol’s criteria were not fully met. Time was always measured by using photocells (Speed Trap II - Browser Timing Systems). The participant was timed from the first cone until they overcame the last cone. Subsequently, participants shot at the goal where two targets were placed, delimited by a rope 1m from each pole. The subjects ended dribbling between the cones at the crescent of the penalty area and shot as quickly as possible without invasion of the area, having 3.65 m for the shot. For efficacy, the ball had to pass between one of the targets set; if it hit the post, it did not count as effective, but if the ball hit the rope, it was considered effective. This procedure was performed 3 times, with 3 min rest between sets. The best time while dribbling between the cones and the effectiveness of the 3 shots were recorded (Figure 1).

### Statistical Analyses

The analysis of all data was performed using processing and statistical analysis software “Statistical Package for the Social Sciences, SPSS Science, Chicago, USA” version 19.0. An exploratory analysis of all data was performed to characterize the values of different variables in terms of central tendency and dispersion. Thus, all variables were subjected to a graphic observation in order to detect the existence of outliers and possible insertion of inaccurate data. In the statistical analysis, the averages and the respective standard deviations of each variable in the study and in all contexts of planned analysis were calculated. Aiming to perform inferential statistical analysis, it was necessary to assess the normality of distribution of the data collected. Therefore, and taking into account the biological nature of the data, an analysis of the distribution type was made by using the Shapiro-Wilk test. The homogeneity of variance and covariance were tested with the Levene’s test and sphericity with the Mauchly test. Once verified the assumptions of the usage of parametric tests, one-factor analysis of variance (ANOVA) was used to compare the mean values of the variables in study. When this procedure was complete and found that there were no statistically significant differences, a repeated measures ANOVA was used (2 times × 3 groups). The significance level was maintained at 5%.

## Results

ANOVA did not identify significant differences between GCT1 and GCT2. However, significant differences were observed in shooting efficiency for those who had had one or two CXT sessions (GCT1 and GCT2) comparing to CONT (F = 1.139; p = 0.02; μ_p_^2^ = 0.531). ANOVA did not indicate significant differences for the following variables: 15 m sprint, 15 m agility with the ball and crossing efficiency. The mean values at the pre-test, post-test and the difference between the pre- to post-test during the study period are shown in Table 2.

## Discussion

The main finding of this study was that shooting efficiency significantly improved in both experimental groups. These results can be explained by the fact that the motoneurons firing rate improved and increased neural coordination (Docherty et al., 2004; Weber et al., 2008). The greater effectiveness of contractile activity may be related to neural adaptations that occurred during the implementation of CXT. In fact, CXT provides an energy transfer between the concentric and eccentric muscle action phases, which gives a positive transfer of muscle demands, better coordination and synchronization of active muscle groups in order to improve and enhance motor skills (Cronin et al., 2001; Robbins, 2005). The improvements in performance with CXT exist when there are mechanisms that trigger post activation potentiation over the mechanisms of fatigue. In contrast, if the mechanisms of fatigue overlap, adaptation will not take place as it occurred in the present study with speed and agility variables. The post activation potentiation is associated with two fundamental conditions, the use of high intensity and short duration contractions, and the recovery intervals between consecutive contractions (Batista et al., 2007). In turn, these two conditions induce often overlapping fatigue (Kilduff et al., 2007). Possibly this study’s participants did not reach balance between the empowerment and fatigue, and therefore, fatigue may have contributed to reduced extent of potentiation.

Another possible justification for the increase in shooting efficiency can be related to coordination gains that the exercise provided. Recently Tessitore et al. (2011) identified significant improvements in coordination in 16 semi-professional soccer players (22.0 ± 3.6 years), after 5 CXT sessions. Like in the present study, participants also performed three specific soccer tests, namely dribbling speed, shooting a motionless ball and shooting from a pass, coordination (lasting 60 s) and flexion and extension of the foot to an increasing speed of execution (80, 120, and 180 b·min^−1^). As a result of this protocol, athletes improved their dribbling speed (p = 0.03), and shooting from a pass (p = 0.02).

In the present study, no significant differences in crossing efficiency were noted. Since the numbers of attempts for crossing were the same as shooting, the results suggest that crossing efficiency is more complex than shooting (e.g., greater distance between the execution of the technical skill and the target area of the ball). Given the impossibility of controlling potential confounding variables in these tasks, this last statement is purely speculative.

The present study did not identify significant differences in terms of speed. The possible causes for not observing significant increments in the 15m sprints may be related to interindividual variability in the sprint technique of the participants of this study and the time of intervention of this study was not enough to observe increments in this capacity. In a study of youth rugby athletes (n = 11; 20.9 ± 3.1 years), where a CXT program was implemented with 5 sessions of 3-RM of the back squat, the authors also noted a lack of significant improvements in terms of maximum speed, controlled by a 30 m sprint (Comyns et al., 2010). In contrast, other studies have found significant speed improvements after CXT (Kotzamanidis et al., 2005; Maio Alves et al., 2010), but with a different protocol and participant ages. Kotzamanidis et al. (2005) identified a reduction of 0.25 s in soccer players in the 30 m sprint, after combining loads between 3-RM and 8-RM in the half squat, step up and leg curl exercises with 4–6 sets of a 30 m sprint. However, the training program used by Kotzamanidis et al. (2005) contained three more training weeks compared to this study. Maio Alves et al. (2010) found significant reductions in a 5 m sprint and a 15 m sprint: 9.2-6.2% in the group that performed 2-weekly CXT; and 7.0-3.1% in the group that performed 1-weekly CXT. However, participants in the Maio Alves et al.’s (2010) study had an average age (17.4 ± 0.6 years) higher than the subjects in the present study (14.07 ± 0.7 years). This suggests that one possible reason for the absence of significant differences in this study variable can be related to a lack of maturity in coordination. At these ages (14–16 years), the boys are during puberty. It is at this stage that they have a quick increase in height and muscular development. This fast increase affects motor coordination because the capacity in the recruitment of motor units, the intra and inter muscular coordination to the function and the neural control of the movement are affected by the difficulty that they have to adapt to the constant modification of their anthropometric structure.

The improvements in performance resulting from CXT exist when there are mechanisms that trigger the post-activation potentiation over the mechanisms of fatigue. In contrast, if the mechanisms of fatigue overlap, the effects will not be allowed, as it seems to have happened in the present study with the speed and agility variables. The post-activation potentiation is associated with two fundamental conditions, the use of high intensity and short duration contractions, and the recovery intervals between consecutive contractions (Batista et al., 2007). In turn, these two conditions induce often overlapping fatigue (Kilduff et al., 2007). Possibly this study’s participants did not reach balance between the empowerment and fatigue, and therefore fatigue may have contributed to reduced extent of potentiation.

In the present study no significant results in agility were noticed. It is possible that agility training must be performed in a specific environment and independent from speed training programs. Agility movements are more dependent on factors of motor control, rather than maximal strength or muscle power, and this factor may partly explain a lack of significant results at the level of agility (Little and Williams, 2005; Tricoli et al., 2005; Young et al., 2001; Young, 1988). Young et al. (2001) carried out a study including 36 adult males, divided into 3 groups (speed training group, agility training group and control group), with the objective of identifying straight line speed training transfer to agility with various levels of complexity, and the transfer of agility training to motor speed performance. They observed no significant improvements in any of the tests for the control group, the speed group improved significantly in the straight sprint test and in the test of changes of directions; the agility group improved significantly in all the tests with changes of directions, but not in straight line sprinting. The authors concluded that the more complex task of agility training the lower the transfer for speed. The straight line speed and agility training methods are specific, thereby making the transfer limited.

Finally, despite the high intensity and the combination of a number of complex tasks in a single session, complex training showed to be safe among youth soccer players.

## Practical Applications

The addition of CXT to regular soccer training proved to be an effective method in improving physical fitness and motor skills of youth athletes, particularly in increasing shooting efficiency.

Since we did not observe a decrease in performance of the tests of straight line sprinting, sprinting with changes of directions and crossing we can state that CTX may be an important strategy for time management of training allowing the practice of different skills during the same session.

## Figures and Tables

**Table 1:** Sample characterization at baseline.

	GCT1 (n=5)	GCT2 (n=5)	CONT (n=6)
Age	13.80±0.84	14.20±0.45	14.20±0.84
IMC	18.98±1.88	20.38±1.84	18.82±0.66
%MG	12.48±1.54	10.42±2.66	10.94±1.18

(IMC) – Body Mass Index; (%MG) – Estimated Body Fat; GCT1 – Group with one CXT session; GCT2 – Group with two CXT sessions; CONT – Control group

**Table 2:** Exercise performance averages, maximum speed at 15 m (S15), agility with the ball (AG15), crossing efficacy (CE) and shooting effectiveness (SE) between moments (pre- vs. post-test) in the different groups.

	GCT1 (n=5)			GCT2 (n=5)			CONT (n=6)			μ_p_^2^ MxG

	Pre-test	Post-test	Δ	Pre-test	Post-test	Δ	Pre-test	Post-test	Δ	
S15 (s)	2.72±0.25	2.59±0.18	− 0.13	2.60±0.14	2.46±0.15	− 0.14	2.68±0.19	2.66±0.18	− 0.02	0.350
AG15 (s)	10.64±1.82	9.80±1.43	− 0.84	9.64±1.23	8.54±0.37	− 1.10	9.88±0.48	9.90±0.46	0.02	0.240
CE (n)	1.00±0.71	2.20±0.45	1.20	1.00±0.71	2.60±0.55	1.60	0.80±0.84	1.20±0.84	0.40	0.130
SE (n)	1.00±0.71	1.60±0.55	0.60	0.60±0.89	2.20±0.84	1.60	0.80±0.84	0.60±0.55^*^	− 0.20	0.531

* Significant different from GCTI and GCT2 post-tests (p<0.05).

Note: Average values and standard deviations;

Δ – Difference between pre- to post-test; μ_p_^2^ – Partial Eta Square ; M – Moment; G – Group; GCT1 – Group with one CXT session; GCT2 – Group with two CXT sessions; CONT – Control Group.

## References

[b1-jhk-43-105] Batista MA, Ugrinowitsch C, Roschel H, Lotufo R, Ricard MD, Tricoli VA (2007). Intermittent exercise as a conditioning activity to induce postactivation potentiation. The Journal of Strength & Conditioning Research.

[b2-jhk-43-105] Comyns TM, Harrison AJ, Hennessy LK (2010). Effect of Squatting on Sprinting Performance and Repeated Exposure to Complex Training in Male Rugby Players. The Journal of Strength & Conditioning Research.

[b3-jhk-43-105] Cronin J, McNair PJ, Marshall RN (2001). Velocity specificity, combination training and sport specific tasks. Journal of science and medicine in sport / Sports Medicine Australia.

[b4-jhk-43-105] Christou M, Smilios I, Sotiropoulos K, Volaklis K, Pilianidis T, Tokmakidis SP (2006). Effects of Resistance Training on the Physical Capacities of Adolescent Soccer Players. The Journal of Strength & Conditioning Research.

[b5-jhk-43-105] Docherty D, Robbins D, Hodgson M (2004). Complex Training Revisited: A Review of its Current Status as a Viable Training Approach. Strength & Conditioning Journal.

[b6-jhk-43-105] Kilduff LP, Bevan HR, Kingsley MI, Owen NJ, Bennett MA, Bunce PJ, Cunningham DJ (2007). Postactivation potentiation in professional rugby players: optimal recovery. The Journal of Strength & Conditioning Research.

[b7-jhk-43-105] Kotzamanidis C, Chatzopoulos D, Michailidis C, Papaiakovou G, Patikas D (2005). The effect of a combined high-intensity strength and speed training program on the running and jumping ability of soccer players. The Journal of Strength & Conditioning Research.

[b8-jhk-43-105] Kraemer WJ, Fry AJ, Maud, Foster Peter J, Carl (1998). Strength testing: Development and evaluation of methodology. Physiological Assessment Of Human Fitness.

[b9-jhk-43-105] Little T, Williams AG (2005). Specificity of acceleration, maximum speed, and agility in professional soccer players. The Journal of Strength & Conditioning Research.

[b10-jhk-43-105] Maio Alves JMV, Rebelo AN, Abrantes C, Sampaio J (2010). Short-Term Effects of Complex and Contrast Training in Soccer Players’ Vertical Jump, Sprint, and Agility Abilities. The Journal of Strength & Conditioning Research.

[b11-jhk-43-105] McCambridge TM, Stricker PR (2008). Strength training by children and adolescents. Pediatrics.

[b12-jhk-43-105] McGregor SJ, Nicholas CW, Lakomy HK, Williams C (1999). The influence of intermittent high-intensity shuttle running and fluid ingestion on the performance of a soccer skill. J Sports Sci.

[b13-jhk-43-105] Robbins DW (2005). Postactivation Potentiation and Its Practical Applicability. The Journal of Strength & Conditioning Research.

[b14-jhk-43-105] Santos EJAM, Janeira MAAS (2008). Effects of Complex Training on Explosive Strength in Adolescent Male Basketball Players. The Journal of Strength & Conditioning Research.

[b15-jhk-43-105] Summers RB (1999). Complex Training at Ponderosa High. Strength & Conditioning Journal.

[b16-jhk-43-105] Tessitore A, Perroni F, Cortis C, Meeusen R, Lupo C, Capranica L (2011). Coordination of Soccer Players During Preseason Training. The Journal of Strength & Conditioning Research.

[b17-jhk-43-105] Tricoli V, Lamas L, Carnevale R, Ugrinowitsch C (2005). Short-term effects on lower-body functional power development: weightlifting vs. vertical jump training programs. The Journal of Strength & Conditioning Research.

[b18-jhk-43-105] Weber KR, Brown LE, Coburn JW, Zinder SM (2008). Acute Effects of Heavy-Load Squats on Consecutive Squat Jump Performance. The Journal of Strength & Conditioning Research.

[b19-jhk-43-105] Young WB, Mcdowell MH, Scarlett BJ (2001). Specificity of Sprint and Agility Training Methods. The Journal of Strength & Conditioning Research.

[b20-jhk-43-105] Young WB, Jenner A, Griffiths K (1988). Acute enhancement of power performance from heavy load squats. The Journal of Strength & Conditioning Research.

